# Optimization, Purification, and Starch Stain Wash Application of Two New *α*-Amylases Extracted from Leaves and Stems of* Pergularia tomentosa*

**DOI:** 10.1155/2017/6712742

**Published:** 2017-12-17

**Authors:** Imen Lahmar, Hanen El Abed, Bassem Khemakhem, Hafedh Belghith, Ferjani Ben Abdallah, Karima Belghith

**Affiliations:** ^1^Laboratory of Plant Biodiversity and Dynamics of Ecosystems in Arid Environment, Faculty of Sciences, University of Sfax, Sfax, Tunisia; ^2^Laboratory of Plant Biotechnology Applied to Crop Improvement, Faculty of Sciences, University of Sfax, Sfax, Tunisia; ^3^Laboratory of Molecular Biotechnology of Eukaryotes, Center of Biotechnology, Sfax, Tunisia

## Abstract

A continuous research is attempted to fulfil the highest industrial demands of natural amylases presenting special properties. New *α*-amylases extracted from stems and leaves of* Pergularia tomentosa*, which is widespread and growing spontaneously in Tunisia, were studied by the means of their activities optimization and purification. Some similarities were recorded for the two identified enzymes: (i) the highest amylase activity showed a promoted thermal stability at 50°C; (ii) the starch substrate at 1% enhanced the enzyme activity; (iii) the two *α*-amylases seem to be calcium-independent; (iv) Zn^2+^, Cu^2+^, and Ag^2+^ were considered as important inhibitors of the enzyme activity. Following the increased gradient of elution on Mono Q-Sepharose column, an increase in the specific activity of 11.82-fold and 10.92-fold was recorded, respectively, for leaves and stems with the presence of different peaks on the purification profiles.* Pergularia* amylases activities were stable and compatible with the tested commercial detergents. The combination of plant amylase and detergent allowed us to enhance the wash performance with an increase of 35.24 and 42.56%, respectively, for stems and leaves amylases. Characterized amylases were reported to have a promoted potential for their implication notably in detergent industry as well as biotechnological sector.

## 1. Introduction

Maltogenic amylases are widely distributed in microorganisms, plants, and higher organisms and constitute a subfamily of amylolytic enzymes [1, 2]. Through their transglycosylation activity, they were responsible for the solubility increase, the oxidative stability, the sweetness, and the carcinogenicity decrease [3, 4]. Belonging to this enzyme family, *α*-amylases (1,4-a-D-glucan glucanohydrolase) catalyze the hydrolysis of *α*-1,4 glycosidic linkage in starch and related polysaccharides. They represent approximately 25% of industrial enzymes in the global market [5]. Hence, they have an industrial importance which is intensified by their thermal resistance and adaptation to special processes as brewing and liquefaction process [6], paper and textile sectors [7], and heavy-duty and dishwashing detergents [8]. Currently, *α*-amylases were implied also in chemistry, clinical, pharmaceutical, or analytical process [9]. 

From the worldwide enzyme manufacturing, *α*-amylases are included in about 30% of the global detergent industries and in 90% of the solid-liquid laundry [10, 11]. Despite the continuous need of the discovery of new enzymes suitable for new detergent formulations, microbial amylases still have the crucial interest [12–15]. A few researches focused on plants amylases quite useful for cleaning purposes, such as the case of immobilized *α*-amylase soybean onto chitosan enhancing the removal of starch stains [16]. With the ignorance of their local endogenous applications [17], the need to characterize plant amylases by means of their stability, specificity, optimal activity range, and metal dependence still remains.

The fact that *α*-amylases are commonly extracted and purified from different plant organs, such as tubers [18], leaves [19], seeds [20, 21], and stems [22], encourage us to focus on the plant source and mainly wild plants which are not studied in the literature and which may present interesting specific and unique properties. While plants have been considered as a source of abundant enzymes which were well searched in food sectors [23], less attention has been paid to species which can be included in industrial starch processing like* Triticum aestivum, Manihot esculenta,* and* Zea mays* [24].

Asclepiadaceae family was characterized by the richness of several species in different enzymes.* Asclepias fruticosa* contains asclepain in its latex [25] and calotropain was discovered in* Calotropis procera* [26]. A cysteine protease hydrolysing the fibrinogen was found in* Pergularia extensa* and* Cynanchum puciflorum* [27]. Belonging to this family,* Pergularia tomentosa* has gained a recent scientific importance due to its proteinases, rennin, polyphenol oxidase, tyrosinase, *β*-amylase, lipase, L-asparaginase, and lipoxygenase widespread in the latex compared to the crude extract of the whole plant [28–30]. According to the literature, this rangeland species of* Pergularia* contains several secondary metabolites, antioxidative bioactive molecules, and potent antifungal compounds against* Fusarium oxysporum *f.sp.* lycopersici* [31, 32]. It was used as a remedy in traditional medicine in case of tuberculosis, skin diseases, and constipation [33]. It presented anti-inflammatory [34], antidermatophytic [35], molluscicidal [36], and antimicrobial activities [37, 38] and it was known as anticancer agent [39] and insect repellent [40].

In view of the above, the present study reports the purification of *α*-amylases extracted from leaves and stems of the wild medicinal plant,* Pergularia tomentosa*. Several conditions were also optimized such as pH, temperature, and substrate concentration. Additional ions in the reaction medium were classified as activators or inhibitors to achieve high and better enzyme activity.

We also intended to evaluate the stain remover potential of extracted enzymes view of the high detergent industry requisition of amylases using low-cost raw materials. The main purpose of the enzyme application raised in this study is to investigate the cleaning efficiency of plant amylase combined to laundry detergent and its distinctive compatibility as a highly efficient new additive.

## 2. Materials and Methods

### 2.1. Plant Material

*Pergularia tomentosa* L. was identified and collected from the surrounding of Bir Ben Ayed (south of Sfax, Tunisia) [31]. Stems and leaves were rinsed carefully with distilled water to be purified from soil and air environmental impurities. Plants were deposited onto filter paper until relative drying after the rinsing step. Each organ was ground separately in the minimum of distilled water. The mixture was centrifuged at 5000 ×g for 30 min and the obtained supernatant was filtered through the filter paper (150 nm, 5B, Advantec Tokyo, Japan) to obtain a clear crude extract.

### 2.2. Precipitation of *α*-Amylase and Enzyme Assay

Protein precipitation with ammonium sulphate at 80% was carried out with a continuous gentle stirring in ice bath and was stored later overnight at 4°C [41]. The solution was centrifuged at 12,000 ×g for 30 min and at 4°C. The obtained precipitate was dissolved in distilled water and dialyzed against the same solvent for 24 h at 4°C and by changing the solvent thrice. Dialysis was carried out using cellulose tubing (molecular weight cut-off 13,000 kDa, Himedia LA393-10 MT).

The protein content was determined by the Bradford method [42] referring to bovine serum albumin. *α*-Amylase assay was carried out following the DNS (2-OH-3.5-dinitrobenzoic acid) method of Miller [43]. Optical density was measured at 550 nm against substrate and enzyme blank. One unit of amylase was defined as the enzyme amount which releases 1 *μ* mole of glucose per minute.

### 2.3. Effect of pH and Temperature

pH optimum was determined by incubating the amylase-substrate reaction for 10 min at different pH ranging from 4.0 to 10.0. Temperature optimization of amylase was determined by carrying the reaction mixture for 10 min (40–60°C) and keeping a constant pH [44]. pH stability was studied by preincubating 0.5 mL of enzyme with 0.1 M buffer at different pH values for 3 h at 4°C [45]. The thermal stability of *α*-amylase was tested by incubating the enzyme for 3 h at the determined pH optimum and at 50 and 60°C. Samples were withdrawn every 15 min and residual activity was determined.

### 2.4. Effect of Substrate Concentration and Metal Ions

*Pergularia tomentosaα*-amylases activity was determined at several starch concentrations at the range of 1 to 2.5% and dissolved in 0.1 M buffer at the pH optimum. The maximum activity was taken as 100% and relative activity was plotted against different concentration values.

Ca^2+^, Mg^2+^, Cu^2+^, Fe^2+^, Mn^2+^, Zn^2+^, Fe^3+^, and Co^2+^ at 1 and 5 mM were supplemented in the reaction medium of enzyme extract and starch solution and incubated for 30 min at pH and temperature optimum of each plant organ. The enzyme activity without addition of any ions (inhibitor or activator) was considered as 100%.

### 2.5. Purification of *α*-Amylase

The dialyzed fraction was heated at 60°C in water bath for 15 min and the denatured protein precipitate was removed by centrifugation, while the supernatant was checked for activity. Obtained active fraction was loaded onto a Mono Q-Sepharose column (2.1 × 24 cm) preequilibrated with 6.5 mM sodium phosphate buffer (pH 5.0) at 4°C. The same buffer was used to wash the column. Bounded proteins were eluted by a linear gradient of NaCl (0-1 M) in the same buffer at a flow rate of 5 mL min^−1^. Protein content was determined at 280 nm according to the method of Bradford [42]. Amylase activity of the recovered fractions was determined following the DNS method [43].

### 2.6. Compatibility of Stems and Leaves *α*-Amylases with Commercial Detergents

The compatibility of stems and leaves *α*-amylases with commercial available laundry detergents, Persil, Tide, and Savex, was determined. Detergent solutions with a concentration of 7 mg/mL were boiled for 90 min to inactivate any enzyme activity included on their formulation. Cooled solutions were mixed separately with each amylase (1 : 1) and incubated at 50°C for 1 hour. The residual activity was calculated in comparison with the control (instead of the detergent solution).

### 2.7. Efficiency of Stems and Leaves *α*-Amylases in Starch Stain Wash

Wash efficiency of starch stains was studied in the presence of Savex detergent and the two* Pergulariaα*-amylases [46]. White cotton cloth pieces stained with starch solution (0.5%) were placed at 80°C for 30 min to assume the firm binding of stains to the material support. Washing performance was tested by varying the cleaner, as water, water + detergent, water + enzyme, water + detergent (7 mg/mL) + enzyme. Stained cotton cloth piece was incubated in the presence of the corresponding cleaner mixture on a shaker platform (100 rpm) for 30 min at 50°C. Obtained solution was collected for each mixture to measure the concentration of reducing sugars released from starch [43]. The blank consists in distilled water instead of wash liquid. The same assay procedure was followed for stems and leaves *α*-amylases.

The efficiency of starch removal by the washing process was expressed as the following equation [47]:

(1) $$\begin{array}{c} Efficiency\%=\frac{100*A*0.9}{B}, \end{array}$$

where *A* is the amount of glucose released (g/mL) during the wash procedure and *B* is the amount of starch (*μ*g/mL) used for staining the cotton cloth piece.

### 2.8. Statistical Analysis

Data were expressed as mean ± standard deviation and comparisons were made with appropriate controls using Student's *t*-test. Confidence limits were set at *p* < 0.05 for all values analyzed in triplicate.

## 3. Results and Discussion

### 3.1. Optimization and Characterization of *α*-Amylases

**Figure 1:**
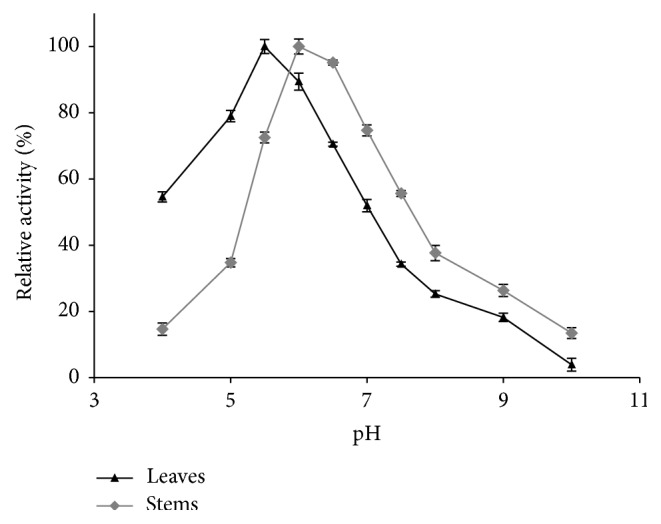
Effect of pH on the activity of *α*-amylases extracted from leaves and stems.

The highest amylase activity of* Pergularia tomentosa* was exhibited at pH 5.5 in case of leaves and at pH 6.0 for the stems (Figure 1). Between pH 4.0 and 6.5, the relative activity of stems amylase retained more than 60% of the maximal activity. However, in case of leaves, 60% of retained activity was observed at the range of pH 5.0–7.0. Beyond pH 8.0, the amylase activity loss was 68% of the initial relative activity. It may be due to the pH effect on the ionization of the group of lateral chains maintaining the enzyme structure and its influence on the active site activities. The pH optimum of amylase extracted from germinated seeds of* Glycine max* is similar to our studied stems [48], while leaves *α*-amylase presented the same pH of* Carthamus tinctorius* amylase isolated from seeds [49].

**Figure 2:**
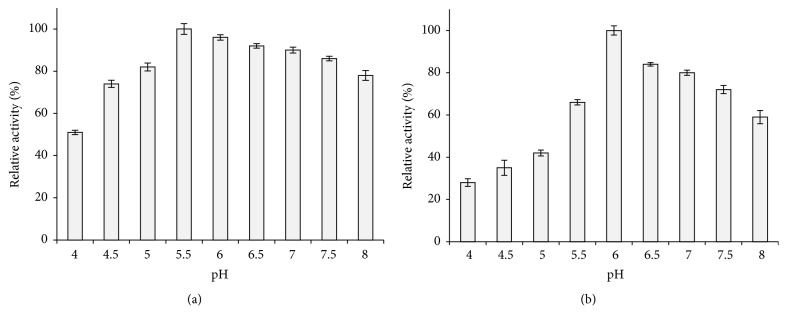
pH stability of *α*-amylases extracted from leaves (a) and stems (b).

Enzymes were incubated for 3 h in several buffers; more than 80% of leaves enzyme activity was retained between pH 5 and 8, suggesting that it was very stable despite the high pH (Figure 2(a)). Compared to the process of leaves amylase, a considerable loss of activity was observed in acidic pH for stems enzyme (Figure 2(b)). The latest amylase was kept stable in the pH range 6–8 and retained approximately 60% of the initial activity after 180 min of incubation.

**Figure 3:**
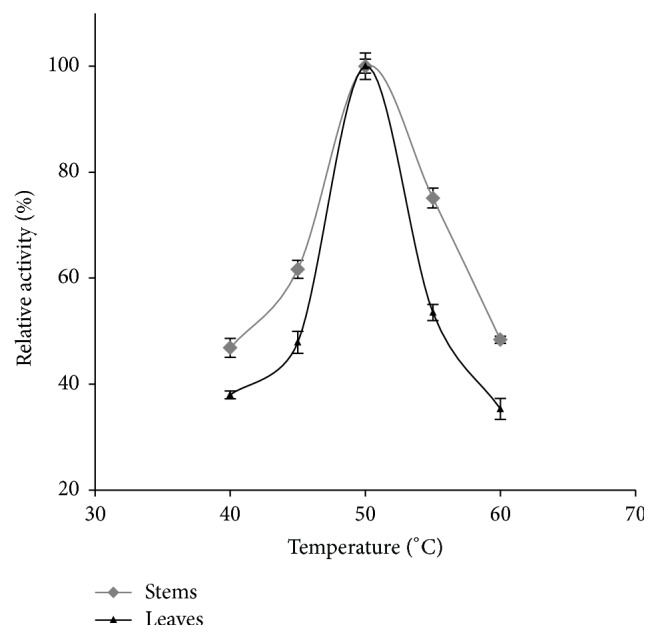
Effect of temperature on the activity of *α*-amylases extracted from leaves and stems.

The curves of the amylase activities as a function of the temperature looked bell-shaped with an optimum at 50°C (Figure 3). Both of stems and leaves curves coincide at the interval of 47 and 52°C. The increase of the temperature was relatively going with the *α*-amylases activity increase, between 40 and 50°C, as assayed at the pH optimum of each plant organ. Beyond the peak of 50°C, the activity began to decline roughly until the temperature changed from 53 to 60°C. Above 60°C, enzymes still retained more than 60% of their initial activities. The temperature optimum varies among species; furthermore, optimal activity of our identified *α*-amylases was slightly lower than* Vigna radiata* and* Pinus koraiensis* (65°C) [50].

**Figure 4:**
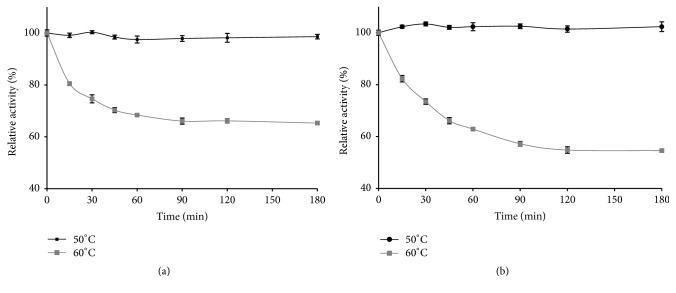
Thermal stability at 50 and 60°C of *α*-amylases extracted from leaves (a) and stems (b).

Thermophilic amylases are mostly searched for starch industries applications [51]. Our leaves amylases are stable at 50°C beyond 150 min and lose just 2% of their initial activity after 90 min of incubation (Figure 4(a)). At 60°C and after 90 min of enzyme incubation, 38% of the initial activity was lost. In the case of stems, 55% of the initial activity remained at 60°C beyond 100 min (Figure 4(b)). The thermal stability of the studied enzymes greatly exceeds the results of Haifeng where the enzyme of* Aureobasidium pullulans* was completely denatured at 60°C after 50 min [52]. This higher percent of the activity retention and thermal stability further encourages the implication of* Pergularia tomentosa* L. in various practical sectors. The observed differences towards the process behavior may be due to the particular genetic heritage of each species [53]. And this thermal stability can be attributed to the presence of some secondary and tertiary binding of the enzymatic proteins enhancing the enzyme structure consolidation and its resistance to the thermal treatment [54].

**Figure 5:**
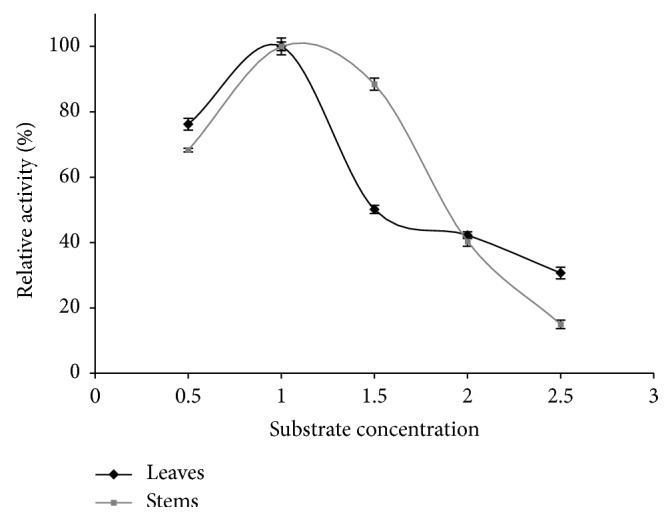
Effect of substrate concentration on the activity of *α*-amylases extracted from leaves and stems.

The effect of substrate concentration was maximal at 1.0% of starch solution in case of leaves and stems (Figure 5). This concentration was also frequently used for amylase assay in previous researches [55, 56]. However, the substrate concentration starting to increase from 0.5% was significantly followed by the enzyme activity increase with enhancement of 23.81 and 31.74%, respectively, for leaves and stems. Then and at 1%, the activity declines gradually, whereas, the shape of the decrease curve for stems was wider in comparison with leaves, especially between the concentrations of range 1–1.5% where there was just 11.56% of enzyme activity lost. The lowering of amylase activity can be explained by the fact that all of the substrate binding sites were filled.

**Figure 6:**
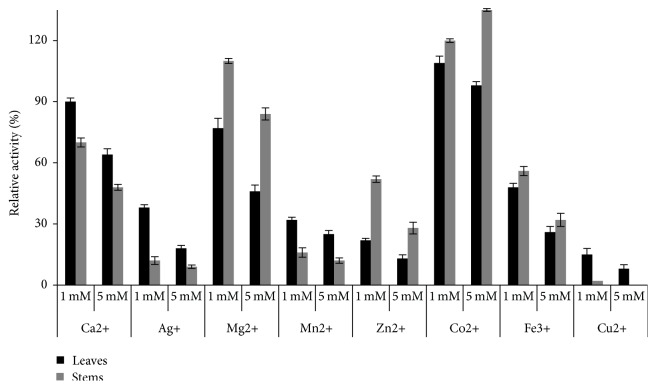
Effect of metal ions on *α*-amylase extracted from leaves and stems.

Leaves *α*-amylase was inhibited by all the tested ions metals, whereas stems *α*-amylase was activated by Co^2+^ with an increase of 35% of the relative activity and it was inhibited by all other metal ions with a variable extent. It was found that calcium has a negative effect, particularly in increasing the concentration. The same process was observed with the amylase of Fenugreek seeds [45]. This effect may be due to the metal competitions and/or to the particularity of the enzyme structure. The known inhibitors Zn^2+^ and Cu^2+^ [57] induce, respectively, a different decrease in enzyme activity at 5 mM. In case of leaves and at 5 mM, Zn^2+^ leads to 87% of activity inhibition and 72% for the stems amylase. At the same concentration, the inhibition of Cu^2+^ was more pronounced and quasi-total. Ag^+^ reported as a strong inhibitor at 2 mM [58] induces a decrease of 82 and 91%, respectively, of amylase from leaves and stems. Thus, among all the metal ions presented in Figure 6, Co^2+^, Mg^2+^, and Ca^2+^ seem the weaker inhibitors of the extracted amylases.

### 3.2. Purification of *α*-Amylases

**Figure 7:**
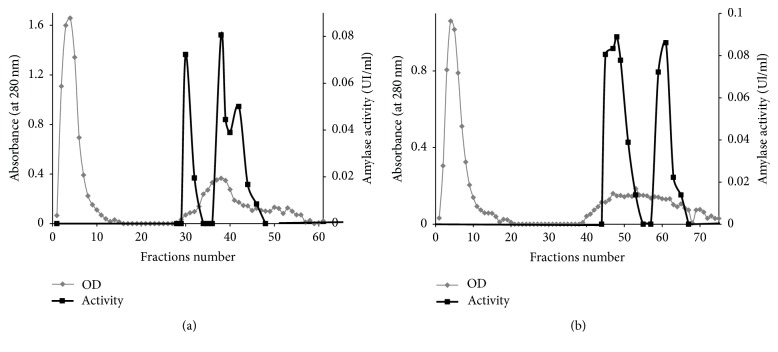
Anion exchange chromatography of *α*-amylase extracted from leaves (a) and stems (b) in Mono Q-Sepharose column eluted with an increased gradient of NaCl.

The purification profiles of *α*-amylases were shown in Figure 7. The anion exchange chromatography of amylase extracted from leaves on Mono Q-Sepharose column eluted with a linear increased gradient of NaCl showed three peaks of activity and just two distinct peaks were revealed in case of stems.

As summarized in Table 1, amylases extracted from the two studied plant organs seemed totally different in view of their specific activities, purification fold, and yield, as well as their profiles after elution with NaCl as shown in Figure 7.

The purification procedure of leaves *α*-amylase leads to a 11.82-fold increase in specific activity for the elution in the range of 25–149 mM NaCl. It was considered as the highest fold in comparison with the two other peaks, while the higher yield of 38.33% was registered for the peak eluted in 225–398 mM NaCl. The main purification fold for stems *α*-amylase was recorded for the peak eluted between 620 and 690 mM NaCl with a yield of 27.27%. Nevertheless, further steps of extract concentration and purification processes such as affinity chromatography could be used to have more pure *α*-amylases from* Pergularia tomentosa*.

### 3.3. Application of Stems and Leaves *α*-Amylases

The above results confirm the largest activity spectra of the two amylases extracted from stems and leaves of* Pergularia tomentosa* at a wide range of pH and temperature. The revealed interesting retained activity at alkaline pH and moderate temperature and the exhibited amylases stability were considered as important criteria during the manufacture of commercial detergents and the degradation of starchy stains residues. The negative effect of calcium on* Pergularia* enzyme activity is searched to fulfil imperfect detergents suffering from oxidants sensitives and calcium-dependent *α*-amylases [59]. Out of the vast pool of microbial amylases enhancing whiteness effect, a similar formulation based on a wild-plant enzyme promotes widespread environmentally safe and low-cost detergents especially in rural areas [60].

However, a lucky inclusion of enzyme in the detergent formulation requires a good compatibility [61]. The data presented in Table 2 showed excellent stability and compatibility of stems and leaves *α*-amylases of* Pergularia tomentosa* with the tested three commercial detergents.

According to the results and compared to the two other detergents, detergent A was considered the lowest compatible with stems and leaves amylases via the obtained residual activities, respectively, 65.22 and 80.5%. Furthermore, leaves amylases seem to be more compatible with detergents A and C than stems amylase with a quasi-compatibility with the detergent B. The detergent C was found to be more compatible with the two amylases, by retaining an enzyme activity of 87.91% and 94.05%, respectively, for stems and leaves.

Application of *α*-amylases is still very limited and a few studies reported the possibility of their implication in wash performance implying their compatibility with detergents, powder as well as liquid. Besides, the obtained results presented in Table 2 could be compared to previous researches yet they focused just on amylases extracted from fungi and bacteria [12, 13]. In the data described above, thermal-stable *α*-amylases from* Pergularia tomentosa *should be suggested as a competitive additive in detergent formulations, while the detergent effect on the residual activity of the discovered amylases may be attributed to the detergent composition [13].

**Figure 8:**
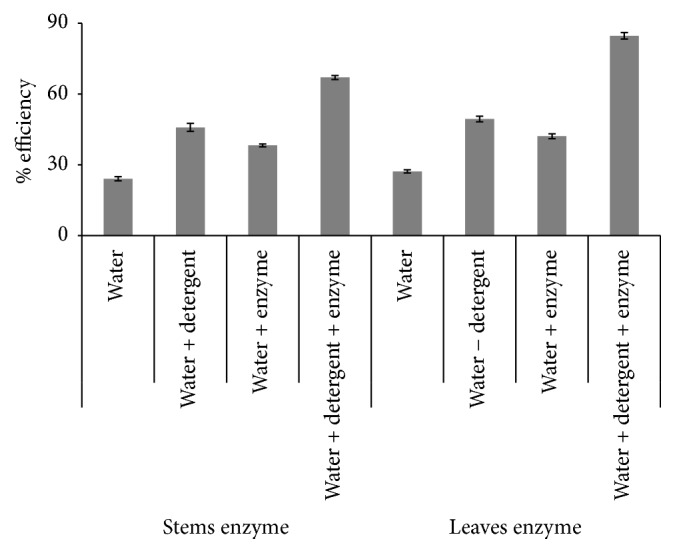
Efficiency of starch stain removal of stems and leaves *α*-amylases.

Previous studies reported the efficiency of bacterial *α*-amylase towards several raw starch sources, for instance, soluble starch, potato curry, corn, and wheat starches [14] as well tomato sauce and egg yolk [15].  Figure 8 reveals that the combination of water, detergent, and *α*-amylase of stems and even leaves of* Pergularia tomentosa* greatly enhances the ability to remove starchy stains from cotton cloth pieces compared to the mere use of detergent or *α*-amylase. The revealed increase of washing efficiency by the supplement of amylases to detergent was in accordance with other studies [14, 47]. In fact, this combination was significantly improved when leaves *α*-amylase was added with an increase of 35.24% and 42.56%, respectively, with the simple enzyme wash and with the detergent wash.

The two* Pergularia tomentosaα*-amylases could be integrated in industrial sectors as catalysts of stains removal and incorporated in different formulations of detergents. It could solve the problems of human skin sensitivity and side effects of the detergents residues evacuated in the environment by decreasing the amount of industrial components like surfactants, bleach, and cobuilders through the challenge of natural *α*-amylases. The reputable thermal stability of* Pergularia* amylases minimising the risk of contamination and the diffusion rate warrants further investigation for further industrial and biotechnological applications with a low-cost of external cooling [62].

## 4. Conclusion

The described work in this paper was attempted to characterize and purify plant*α*-amylases which could be exploited in several fields like the hydrolysis of oil-field drilling fluids and the paper industry. The study of biochemical characteristics of amylases identified from* Pergularia tomentosa* showed a promising range of pH stability and an interesting thermal stability especially at 50°C without requirement of calcium. The simple and cheap extraction procedure of the new stems and leaves *α*-amylases as well as the interesting purification fold and yields raises the great potential of our studied plant in starch stain removal as a source of biological active substances. By immobilization procedure, the amylases properties may be also improved to be implied in successful and modern biotechnology sectors.

## Figures and Tables

**Table 1:** Purification of *α*-amylases extracted from leaves and stems of *Pergularia tomentosa*.

Step	Specific activity (U/mg)	Purification (fold)	Yield (%)
Leaves			
Crude extract	0.547	-	-
Heat treatment	0.558	1.012	76.905
Anion exchange chromatography			
25–149 mM NaCl	6.463	11.821	17.19
225–398 mM NaCl	2.672	4.887	38.333
402–450 mM NaCl	3.787	6.927	23.81

Stems			
Crude extract	0.554	-	-
Heat treatment	0.619	1.117	84.567
Anion exchange chromatography			
290–435 mM NaCl	5.41	9.756	28.224
620–690 mM NaCl	6.048	10.924	27.272

**Table 2:** Effect of different detergents on the residual activity of extracted *α*-amylases from stems and leaves of *Pergularia tomentosa*.

Additive	Residual activity of stems amylase (%)	Residual activity of leaves amylase (%)
Control	100	100
Detergent A	65.22 ± 0.08	80.5 ± 0.93
Detergent B	82.63 ± 1.14	81.80 ± 0.54
Detergent C	87.91 ± 1.5	94.05 ± 1.32
